# A gradient core-shell microsphere to enable effective embolization of liver cancer via interventional therapy

**DOI:** 10.1093/rb/rbag149

**Published:** 2026-07-22

**Authors:** Hongtao Sun, Lijuan Yao, Xin Wang, Hao Xu, Chaozhou Li, Xin Li, Lin Yu, Haibo Che, Peng Sun, Yinghua Zou, Jiandong Ding

**Affiliations:** State Key Laboratory of Molecular Engineering of Polymers, Department of Macromolecular Science, Fudan University, Shanghai 200438, China; Cardiolink Science (Shenzhen) Medical Technology Development Co., Ltd, Shenzhen 518122, China; Cardiolink Science (Shenzhen) Medical Technology Development Co., Ltd, Shenzhen 518122, China; State Key Laboratory of Molecular Engineering of Polymers, Department of Macromolecular Science, Fudan University, Shanghai 200438, China; Interventional Radiology Department, The Affiliated Hospital of Xuzhou Medical University, Xuzhou 221004, China; Cardiolink Science (Shenzhen) Medical Technology Development Co., Ltd, Shenzhen 518122, China; Cardiolink Science (Shenzhen) Medical Technology Development Co., Ltd, Shenzhen 518122, China; State Key Laboratory of Molecular Engineering of Polymers, Department of Macromolecular Science, Fudan University, Shanghai 200438, China; Cardiolink Science (Shenzhen) Medical Technology Development Co., Ltd, Shenzhen 518122, China; Cardiolink Science (Shenzhen) Medical Technology Development Co., Ltd, Shenzhen 518122, China; Department of Interventional Radiology and Vascular Surgery, Peking University First Hospital, Beijing 100034, China; State Key Laboratory of Molecular Engineering of Polymers, Department of Macromolecular Science, Fudan University, Shanghai 200438, China

**Keywords:** polymer, poly(vinyl alcohol), microsphere, embolization, liver cancer, interventional treatment

## Abstract

Embolic microspheres are important for the interventional treatment of solid tumors, but it is a dilemma for microspheres to keep sufficient strength and resilience. Herein, we propose a strategy to fabricate core-shell poly(vinyl alcohol) microspheres with gradient crosslinking (GCL) on an industrial scale to solve the dilemma. The synthesized GCL microspheres exhibit superior mechanical characteristics in comparison to the homogeneous microspheres and the conventional core-shell microspheres produced by standard one-step and two-step methods, respectively. An *in vitro* model is conducted to assess the distribution in the vascular network and migration over time of three microspheres. The GCL microspheres possess optimal strength and flexibility, facilitating distal vascular embolization and reducing the risk of microsphere migration over time. The performances are validated *in vivo* with the porcine renal model in large animal experiments. Clinical trials addressing liver cancer further confirm the embolic efficacy and safety of the GCL microspheres in humans. The relationship between mechanical properties and embolic efficiency offers valuable insights for the development of other embolic agents in the formulism of interventional therapy.

## Graphical abstract

**Graphical Abstract:**
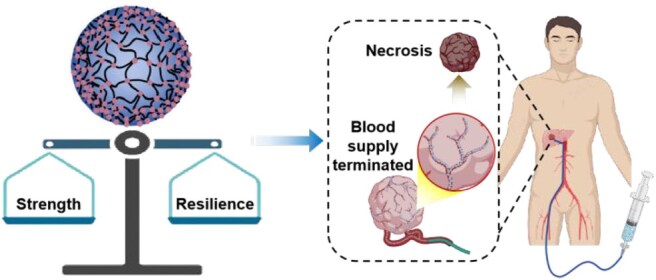

## Introduction

An embolic agent is a key medical device in transarterial chemoembolization (TACE) to treat solid tumors in a minimally invasive manner, particularly appropriate for liver tumor [1, 2]. The TACE technique involves selective insertion of a catheter into the specific artery that supplies blood to the tumor cells and sometimes administer the appropriate amount of chemotherapeutic drugs along with embolic materials at a suitable rate. This process effectively disrupts the blood supply to the tumor, leading to the death of tumor tissue due to ischemia and necrosis [3]. Gelatin sponges or irregular poly(vinyl alcohol) (PVA) particles were initially employed as embolic agents [4]. The commercially available embolic microspheres remain a challenge in achieving a suitable balance between strength and elasticity: if the strength is insufficient, the microspheres are easily broken, constituting an issue for safety; on the contrary, if the strength is excessive, the microspheres become rigid and exhibit reduced elasticity, making it difficult for them to pass through the catheter. Furthermore, they may not align well with the blood vessels, resulting in uneven distribution within the vessels, leading to poor embolization and an increased risk of blood vessel recanalization [5].

**Figure 1:**
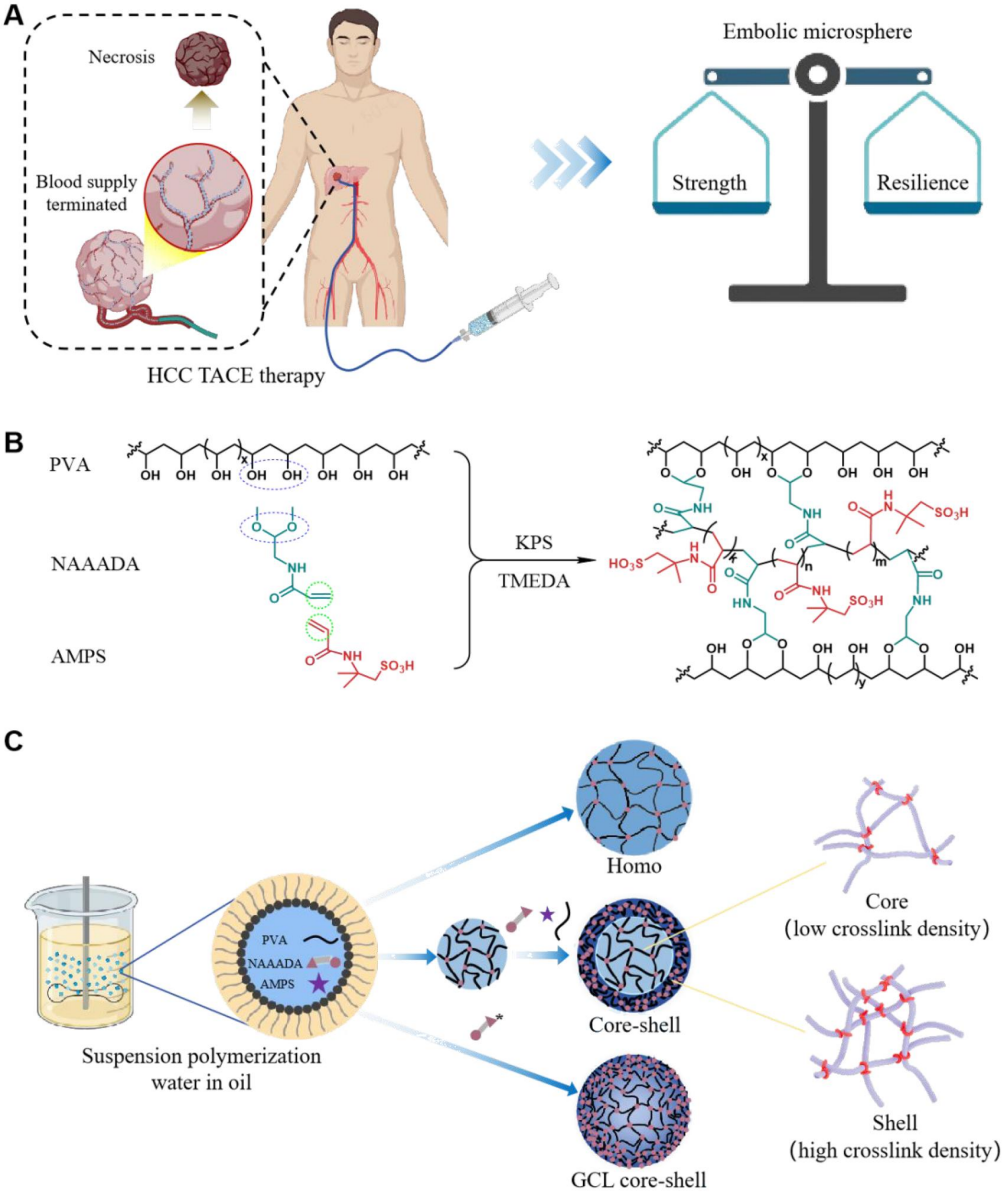
The basic idea of the GCL core-shell embolic microsphere synthesized in this study. (**A**) Illustration of the TACE therapy for hepatocellular carcinoma and the balance of embolic microspheres between strength and resilience. (**B**) Chemical reaction and structure change from starting materials to product microspheres. (**C**) Suspension polymerization (water-in-oil) of microspheres with different crosslink structures based on different synthesis methods, where the crosslink agents denoted as * in the GCL method are introduced in two stages.

To solve the dilemma of strength and resilience of embolization microspheres for interventional treatment, we herein design an embolic PVA microsphere with a core-shell structure through the process of gradient crosslink (GCL; Figure 1). It consists of an outer layer with a high level of crosslink and a core with a low level of crosslink. The former guarantees excellent mechanical strength, making the GCL microspheres resistant to breakage, while the latter enhances its resilience. Furthermore, a self-built *in vitro* vascular system model was introduced to mimic the distribution of microspheres in the desired region. Animal studies confirmed the feasibility of the GCL microsphere in a porcine renal embolization model. The GCL microsphere prepared on an industrial scale was further assessed in clinical trials, indicating the excellent occlusion performance without adverse symptoms of the new microsphere.

## Materials and methods

### Raw materials

The chemical reagents used in this study were purchased from Aladdin.

### Synthesis of homogeneous microspheres

A solution with a concentration of 2 wt.% cellulose acetate butyrate in butyl acetate was first prepared by stirring the mixture under constant heating conditions in a water bath at a fixed temperature of 60°C. This mixture was treated as an oil phase. A 10 wt.% aqueous solution of PVA was prepared at 95°C. 0.8 g of 2-acrylamido-2-methylpropanesulfonate (AMPS), 0.03 g of N-acryloyl-aminoacetaldehyde dimethylacetal (NAAADA) and 0.02 g of potassium persulfate (KPS) were weighed and dissolved in 3 mL of deionized water, respectively. The above mixture was then combined with 20 mL PVA solution to obtain a homogeneous aqueous phase. The aqueous phase was gradually introduced into the oil phase while stirring at a speed of 250 rpm, resulting in the formation of a water/oil reverse polymerization system. Subsequently, 0.05 mL of tetramethyl ethylenediamine (TMEDA) was introduced into the reactor, and the reaction proceeded at a consistent temperature of 60°C for a duration of 6 h.

### Synthesis of conventional core-shell microspheres

The oil phase was prepared as described in homogeneous microspheres, sometimes denoted as homo microspheres. The amount of NAAADA in the aqueous phase was 0.02 g, while the rest was kept the same with homo microsphere. The initial microsphere was obtained by slowly dropping 2/3 volume of the prepared aqueous phase into the oil phase under 250 rpm stirring. Afterwards, TMEDA was added to the reactor to activate the reaction at 60°C for 3 h. The fabricated microspheres were cleaned with ethyl acetate, acetone and deionized water. NAAADA of 0.01 g was thoroughly mixed with the remaining 1/3 of the aqueous phase and placed into the reactor together with the cleaned microspheres generated previously. The secondary reaction, lasting 6 h, was conducted following the addition of TMEDA with constant stirring to form an outer layer on the surface of the initial microsphere.

### Synthesis of gradient crosslink microspheres (GCL microspheres)

The oil phase was generated following the procedure of homo microsphere preparation. The aqueous phase contained 0.02 g of NAAADA, whereas the remaining components were kept unchanged with homo microsphere. The aqueous phase was carefully dripped into the oil phase under 250 rpm mixing. After TMEDA was added, the reaction was activated and lasted for 1 h at 60°C. Another 0.01 g of NAAADA was introduced into the system, lasting for 5 h.

### Post-synthesis treatment of microspheres

Briefly, the synthesized microspheres were rinsed with ethyl acetate and acetone to eliminate any remaining solvent. The microspheres were transferred into sodium bicarbonate solution for neutralization. The microspheres were washed three times with deionized water. The cleaned microspheres were sieved using stainless steel meshes with varying pore sizes. Thus, calibrated PVA microspheres with different particle sizes were obtained and stored in saline.

### Morphology and particle-size measurements

Microscopic analysis was performed using an optical microscope system (VHX-950F, Keyence). The microspheres were also stained by immersing in Congo red solution at a concentration of 5 mg/mL to obtain better visibility for morphology analysis. The excess colorant was removed by rinsing with deionized water. Drain and place the stained microspheres on the glass slide for further assessments. A laser scattermeter (Topsizer, OMEC) was used to obtain the particle-size distribution of the microspheres.

### SEM and element analysis

Scanning electron microscope (SEM) images taken with a microscope Quanta 200 operated in the mode of secondary electron detection with an acceleration voltage of 5.00 kV. Prior to observation, the microspheres were fixed and cut manually. Then the specimen was transferred onto SEM stubs, and their cross-sections were applied with an ultra-thin gold coating by using a sputtering coater prior to analysis. Elemental mapping was performed in a scanning electron microscope fitted with an energy-dispersive X-ray spectroscope of Oxford instruments.

### Water content analysis

The water content of the beads was determined gravimetrically. It was expressed as the difference between the weight of the beads in the hydrated state and the weight of the beads after dehydration. A hydrated state was considered when excess solution was removed and the beads were left as a slurry. Dehydration was carried out by driving off residual water in an oven at 105°C. Initially, this process was carried out for 24 h. After that time, every 8 h the vials were reweighed until no further decrease in mass was observed.

### Mechanical properties

The compression test was performed using a texture analyzer (TX-XTplusC, Stable Micro System) with a 50 mm diameter cylindrical probe (P/30) fitted with a 50 N load cell. First, microspheres were spread on a flat surface to form a monolayer. The microspheres were then compressed 50% with a probe, and the resistance to compression force was calculated. The experiments were conducted at ambient temperature and replicated a minimum of three times to ensure statistically significant findings.

### Suspension tests

For the suspension of microsphere determination, each type of microsphere with a 300–500 μm diameter was prepared. Each sample was mixed using 20 mL syringes and 3-way connectors with saline (0.9% w/v) and a contrast medium (Iohexanol 300) at specific ratios. Shake the syringe vigorously after the three contents are mixed, then start the timer to measure the duration required for the microspheres to achieve the suspension state. Place the syringe in an upright position and record the time it took for the microsphere to remain in suspension.

### Catheter deliverability

For examination of catheter deliverability, microspheres were pulsed-injected into microcatheters of varying sizes through a three-way connector at a rate of 1 mL/min. The smallest size of microcatheter that allowed the passage of microspheres was noted. The microsphere deliverability test was assessed using 1.8 Fr, 2.0 Fr, 2.2 Fr and 2.4 Fr microcatheters, which are commonly used by physicians in clinical practice.

### *In vitro* occlusion performance

Understanding the flow characteristics of embolic agents after they are introduced into the bloodstream is crucial for estimating the potential location of embolic agents inside the tumor blood vessels. This is recognized as a factor that determines the effectiveness of therapy. Therefore, a three-dimensional model simulating the vessel system was designed to evaluate the *in vitro* occlusion performance of microspheres. The stained microspheres were prepared and mixed with the radiopaque contrast medium (Iohexanol 300) before injection. The microsphere suspension was injected in the model slowly. The flow behavior and distribution of microspheres within the lumen could be detected. As the target vessel was blocked, saline was pumped into the feeding artery for 3 h to determine the migration of the embolic microspheres. The photographs at moment 0 and 3 h post-flushing were recorded as well.

### *In vivo* studies of embolization performance in a porcine kidney model

Animal research protocols were reviewed and approved by the Institutional Animal Care and Use Committee (IACUC) of Shenzhen Advanced Medical Services Co., Ltd (Study No. AAS A190804P). The studies were carried out in compliance with ‘Guide for the Care and Use of Laboratory Animals’. Eighteen Bama mini-pigs weighing 20–30 kg were included and randomly divided into two groups. Both the GCL microspheres and the commercialized Embosphere® (Merit Medical, USA) were selected with the same diameter (300–500 μm) for *in vivo* studies.

The animals were fasted 12 h prior to the procedure. Anesthesia was maintained by intramuscular injection of Sutent 50 (15–25 mg/kg), intravenous 6 mg/kg propofol, and then 0.5–2% isoflurane, along with a slow intraventricular saline drip. The femoral artery was punctured using a modified Seldinger puncture procedure, ensuring aseptic conditions. Afterwards, a 5F catheter was inserted into the entrance of the right renal artery under digital subtraction angiography (DSA) monitoring, and high-pressure contrast was employed to verify the target embolic vessel. A 2.7F microcatheter was placed into the lower pole branch arteries of the right kidney. Once the appropriate location was identified, the proportionally mixed suspension of the microspheres and the contrast agent was injected into the target vessel at an average injection rate about 1 mL/min. When all target vessels were occluded, the endpoint of embolization was reached. The dosage of microspheres and the duration of the embolization process were carefully documented. Five minutes following the completion of embolization, the right renal artery was visualized while the target arteries were invisible, indicating that the desired outcome had been achieved. DSA angiography and CT imaging were conducted on Day 2, Day 7 and Day 28 post-embolization to assess the impact of embolization and recanalization on the affected vessels. Three animals were observed in each group at each time point.

### Specimen acquisition and pathological analysis

For blood sample collection and measurement, porcine blood specimens were collected for hematological, coagulation, renal and hepatic function assessment at preoperatively and 2, 7 and 28 days postoperatively. All the animals were fasted overnight before blood collection. In order to anatomy and analysis, the animals were dissected after euthanasia of the animals at each follow-up point. The main organs, including hearts, livers, spleens, lungs, brains and kidneys were taken for gross observation, and abnormalities and lesions were recorded. To enable pathologic analysis, the kidneys were fixed with 10% neutral formalin, then sliced transversely from the upper pole to the lower pole in 1 cm increments to include the upper pole and embolic lower poles. The pathological sections were processed and stained with hematoxylin and eosin (HE). They were then examined and photographed using a microscope. The postoperative outcomes were evaluated by experienced pathologists.

### Clinical case in liver cancer treatments

A prospective, multicenter, randomized controlled clinical trial was conducted to determine the safety and effectiveness of the GCL microspheres for embolization in humans. The clinical trial (protocol number KRCLC04) was approved by the Ethics Committee of Peking University First Hospital. This article reports a clinical implantation in a 51-year-old male patient. He experienced a comprehensive pretreatment evaluation, consisting of a clinical history, contrast-enhanced magnetic resonance imaging (MRI), tumor marker measurement and liver function tests. The pretreatment results revealed multifocal hepatocellular carcinoma (HCC) at Ib stage. Child-Pugh classification was A, and Eastern Cooperative Oncology Group (ECOG) performance status was 0. Therefore, this patient was suitable for TACE treatment.

Dexamethasone and antiemetics were given prior to the TACE procedure. After subcutaneous local anesthesia using 2% lidocaine, the right femoral artery was punctured using the Seldinger technique, and a 5-F vascular sheath and catheter were placed. DSA was performed to clarify the arterial anatomy, tumor blood supply artery and tumor location. The target tumor donor arteries were superselected by microcatheter. Under fluoroscopic observation, lipiodol and epirubicin emulsions were thoroughly mixed by the pumping method and slowly injected into the tumor artery through a microcatheter, followed by the infusion of GCL microspheres. The endpoint of embolization was defined that ≥90% disappearance of tumor staining under DSA monitored. The microcatheters were removed slowly when the TACE procedure was completed.

The follow-up study was conducted for 3 months. The blood routine, blood clotting test and liver function were examined after 1 day and 7 days of operation. The decision of whether to perform further embolization was based on the presence of active lesions by analyzing tumor markers and MRI results. Clinical response was evaluated by MRI according to the mRECIST criteria at 1–3 months after TACE.

### Statistical analysis

The acquired data were statistically compared based on time using a nonparametric method termed the Mann–Whitney *U* test. The statistical analysis was conducted using the Prism software (version 8.0 GraphPad Software). The descriptive data were displayed as mean and standard deviation. A statistical threshold of *P *< 0.05 was considered statistically significant.

## Results

### Fabrication of microspheres

The gradient crosslink method is based on suspension polymerization (Figure 1B). To modify the PVA microspheres, AMPS is selected as the functional monomer, while NAAADA serves as the crosslink agent. This is a free-radical polymerization initiated by an oxidation-reduction reaction. KPS and TMEDA serve as an initiator and an accelerator, respectively. To regulate the chemical structure, the crosslink agent was added into the reaction system twice. As a result of the continuous reaction, the subsequently added crosslink agent was not homogeneously distributed within the microspheres. Eventually, the crosslink agent was more concentrated toward the outer layer, resulting in the formation of a core-shell-like structure in our GCL microspheres as shown in Figure 1C.

We also prepared two control microspheres using alternative synthetic approaches under a given quantity of the crosslinker. The conventional one-step crosslink polymerization was employed to prepare homo microspheres, whereas the naive core-shell microspheres were manufactured using a two-step process, resulting in distinctly different crosslink densities in the core and shell regions. Comparison among the distinct crosslink structures of these manufactured microspheres is helpful for investigating the impact of the crosslinker distribution on the microsphere structure.

### Morphology, size distribution and elemental analysis of microspheres

**Figure 2:**
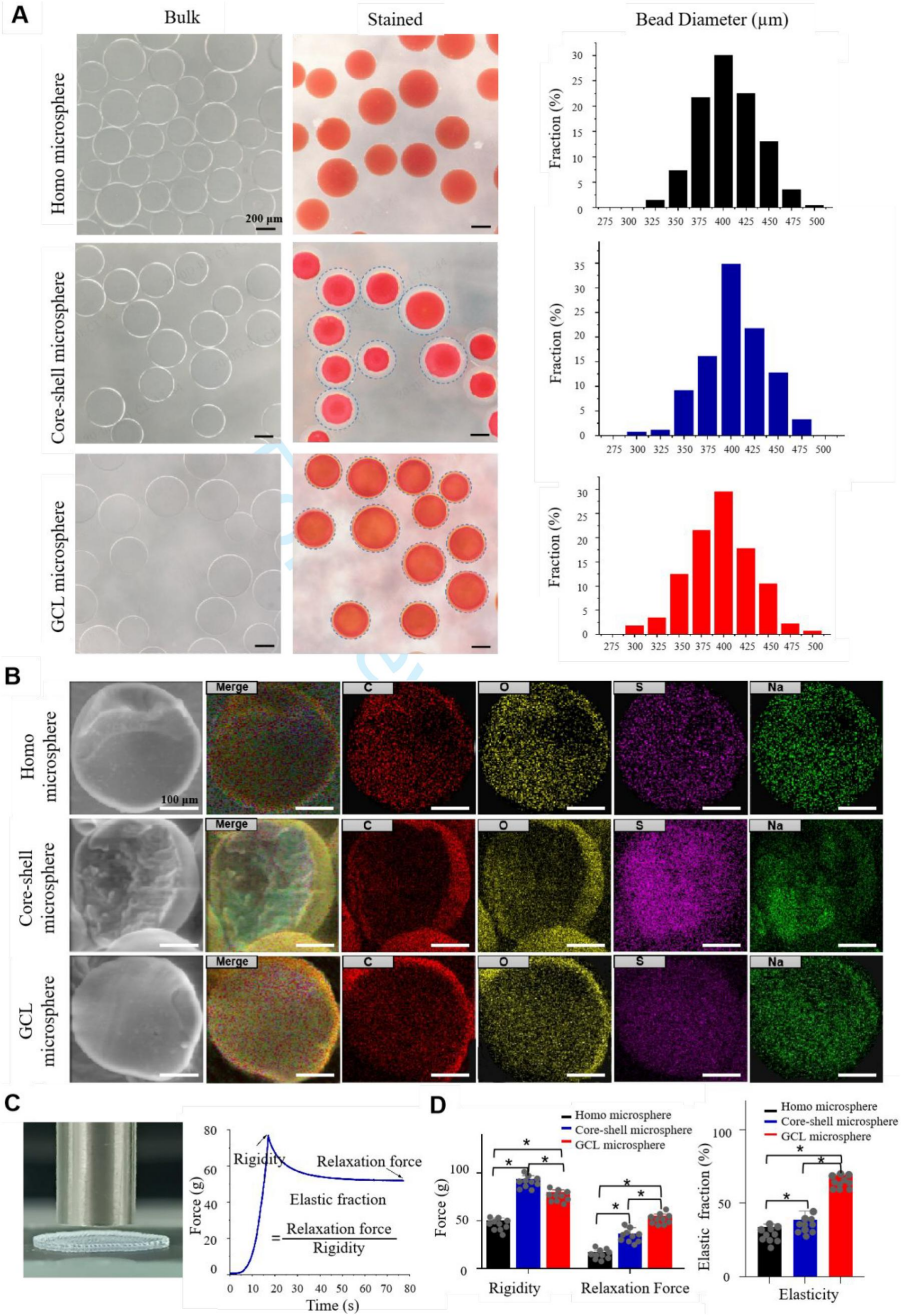
Characterizations of microspheres prepared with different crosslink procedures. (**A**) Morphology analysis of bulk microspheres and dyed microspheres (scale bar 200 μm) and size distribution of 300–500 μm microspheres. Data were normalized by dividing each particle-size range by the total particle-size count. (**B**) Elemental analysis of microspheres with different crosslink structures (scale bar 100 μm). (**C**) Mechanical properties analysis of microspheres. Left is a photograph of a monolayer microsphere before compression for mechanical properties analysis; and the right shows the compression curves of GCL microspheres at 50% deformation. (**D**) Comparative mechanical properties tests of various microspheres. *P < 0.05 (*n *= 10 per group).

The three as-fabricated microspheres were observed with an optical microscope (Figure 2A). All microspheres displayed spherical shapes in the bright-field mode. Different from homo microspheres, the core-shell microspheres produced through two-step synthesis exhibited a clear boundary between the core and shell. The GCL microspheres also showed a color distribution that a shell with high crosslink density is preferentially formed on the outer surface, while the concentration of crosslink agent gradually decreases in the inner layer due to the high diffusion resistance. The microspheres were also detected in a laser particle-size analyzer, and their distributions were found to be similar with diameters of 300–500 μm. For comprehensive investigation of GCL microspheres, diameter distributions of varied sizes prepared by using a series of sieves were detected and are shown in Supplementary Figure S1.

SEM and energy-dispersive X-ray spectroscopy (EDS) analysis of these microspheres were conducted for further understanding of the crosslink structures. The elemental analysis was focusing on carbon (C), oxygen (O), sulfur (S), sodium (Na), as nitrogen concentration was too low to detect (Figure 2B). All four elements were distributed around the cross section of homo microspheres. More of C and O were observed in the outer layer of both the core-shell and GCL microspheres, indicating a more compact shell and a comparatively loose core. This feature is due to the higher abundance of PVA and NAAADA in the outer layer.

### Water content and mechanical properties of microspheres

The crosslink degree of microspheres influences intimately their water content. In general, microspheres with a higher crosslink degree exhibit lower swelling ratios and lower water content. The water content investigation of three groups of microspheres with the same diameter range (300–500 μm) was implemented, and similar water contents were observed (Supplementary Figure S2).

The mechanical properties of microspheres, such as rigidity and relaxation force, determine their deformability and capability to recover their original shape. These parameters play a crucial role in determining the ease of microsphere catheter transit and the effectiveness of embolization [6]. The mechanical characteristics of three microspheres were assessed using a texture tester. A typical curve in Figure 2C demonstrates the compression process of as-prepared microspheres. We suggest that the peak force reflects the rigidity of the microspheres and the endpoint value of the force indicates the relaxation force. The ratio of relaxation force to rigidity is defined as elastic fraction. In this study, the rigidity value indicated the strength of the microspheres, while the elastic fraction represented the resilience of the microspheres after compression.

The three groups of microspheres exhibited significantly different mechanical properties (Figure 2D). Homo microspheres had a low crosslink degree, resulting in low measured rigidity and relaxation force. Thus, homo microspheres were relatively soft and fragile, and breakage might occur when encountering a large external force. Core-shell microspheres represented the highest rigidity value but low relaxation force. This indicated that core-shell microspheres were comparatively challenging to compress, and the recovery rate of deformation was slower once compressed due to their dense shells. GCL microspheres exhibited intermediate rigidity compared to the other two microspheres, and their relaxation force was greater than that of the other two. Consequently, GCL had the highest elastic fraction. The mechanical properties of GCL microspheres well balance strength and resilience: appropriate mechanical strength guarantees the microspheres’ resistance to breakage during compression, while adequate resilience ensures their prompt recovery from compression-induced deformation. Hence, the mechanical characteristics of GCL microspheres provide a strong basis for good embolism performance.

### Suspension property and catheter deliverability of microspheres

**Figure 3:**
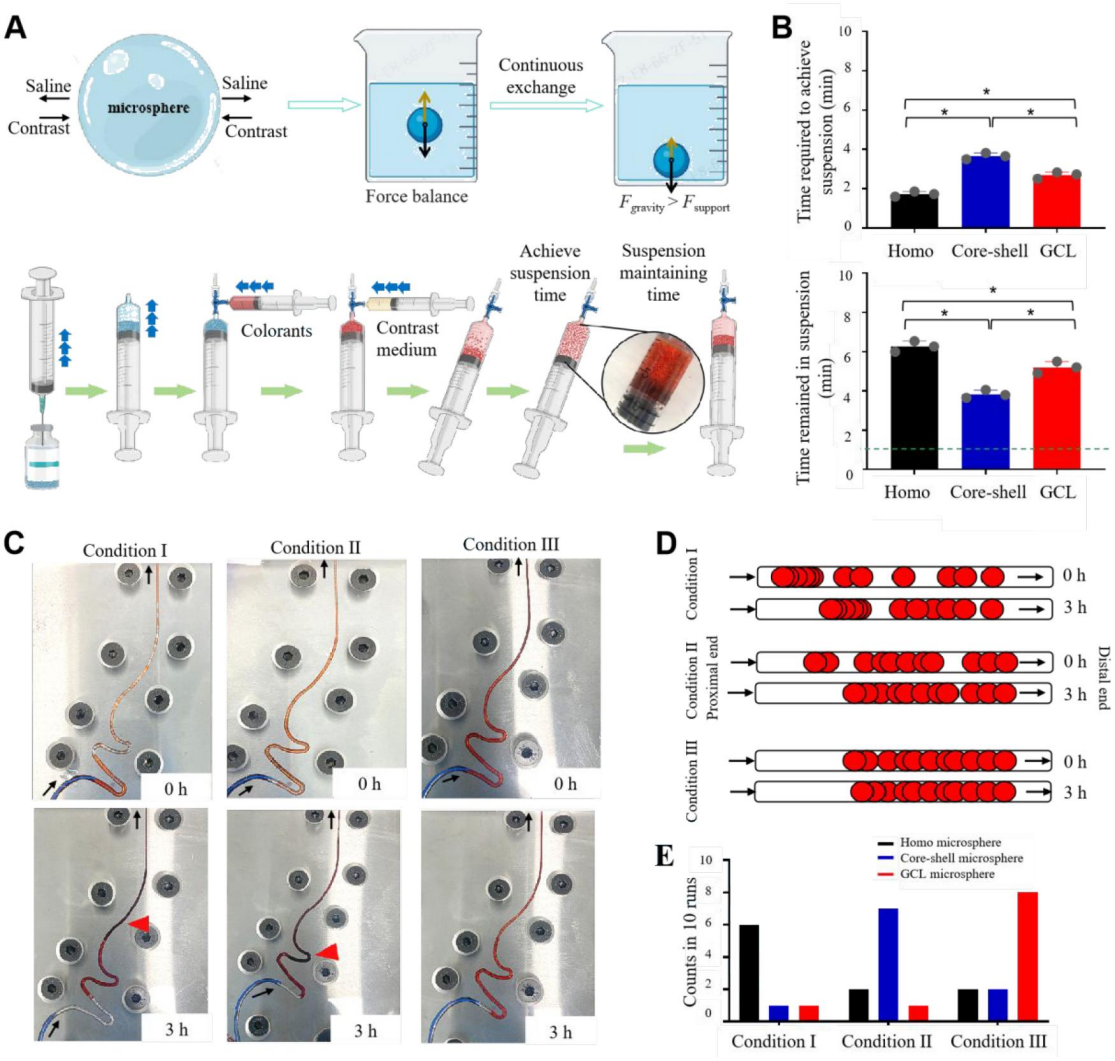
Assessment of suspension ability and catheter deliverability evaluation of microspheres. (**A**) The suspension of microspheres in solution. (**B**) Time required to achieve suspension and suspension-maintaining time for different microspheres (left) and time taken to reach suspension and remain in suspension after mixing with a contrast agent (*n *= 3, *P < 0.05). The dashed line marks 1 min, indicating that the suspension time already meets the requirements for clinical application. (**C**) Three conditions of embolization tests in an *in vitro* model at the immediate point (0 h) and after 3 h of rinsing. (**D**) Schematic illustration of embolization conditions and microsphere migrations, proximal or distal level (close to or distant from the point of introduction into the vessel). In condition I, the microspheres were more concentrated and relatively denser in the proximal vessel. In condition II, the embolic particles exhibited early intermittent embolization, with inadequate occlusion of certain areas of the artery. In condition III, the embolic microspheres were uniformly and systematically positioned within the vessel immediately after the embolism procedure. (**E**) Counting numbers of three conditions (I–III) for three types of microspheres in the *in vitro* embolization model for 10 parallel runs.

In clinical applications, the embolic microspheres are requested to be mixed with the contrast medium, which has greater density than saline for visualization during the surgery process, forming a suspension before infusion (Figure 3A). The physician is interested in two factors regarding the suspension properties of embolic particles: (1) the time required to achieve the suspension state after mixing the microspheres and the contrast medium; (2) the suspension’s maintaining duration. The former influences the preparation time for the procedure, while the latter affects the efficiency of embolization. The quicker the microspheres achieve suspension, the less waiting time for a physician to carry out the treatment. The longer suspension maintaining time the microspheres have, the better embolization the operation gains. The insufficient duration of microspheres might result in two consequences: either there is a shortage of contrast, causing most of the microspheres to settle in the bottom part of the syringe, or there is excessive contrast, causing the microspheres to float in the top section. Both scenarios result in uneven distribution of microspheres in the syringe, leading to accumulation of microspheres in the catheter hub or even the blockage of the catheter, significantly impeding the seamless progression of the surgery.

All three groups of microspheres were mixed with a contrast medium (Iohexanol 300) in a 1:1 volume ratio, which is the typical ratio applied in clinical settings. Then, the suspension properties of three groups of microspheres were evaluated within a 20 mL syringe. According to Figure 3B, when operated with the same dosage of contrast, homo microspheres achieved the suspension state most rapidly, core-shell microspheres exhibited the slowest rate, and GCL microspheres fell in between. The variation was closely linked to the crosslink structure of the three microspheres. Generally, microspheres with lower crosslink degrees allowed for faster penetration of the contrast molecule and were more likely to reach equilibrium. In the comparison of suspension maintenance duration, all three microspheres exceeded 1 min, which are the minimum clinical usage requirements [7–9]. The maintenance time of GCL microspheres was between the homo microspheres and core-shell microspheres.

We also examined the catheter deliverability of microspheres using microcatheters of various diameters. The test followed the conventional approach used by doctors in clinical practice (Supplementary Table S1). Microspheres were mixed with the contrast medium and reached a suspension state prior to the deliverability test. When an easy injection of microspheres via the catheter without any microsphere aggregation or blockage was observed, it was considered that this microsphere system had excellent catheter deliverability for a specific diameter. Homo microspheres and GCL microspheres could pass through microcatheters with minimum diameter 1.8 Fr because of their higher deformability. In contrast, core-shell microspheres had slight resistance and occasional clogging when passing through 1.8 Fr microcatheters due to their dense outer layer and only passed through the diameter above 2.2 Fr microcatheters smoothly. Following the delivery, a visual assessment was carried out on all microspheres, revealing that all delivered samples maintained their spherical shape without any signs of fragmentation. Briefly, homo microspheres and GCL microspheres exhibited lower probability for catheter occlusion in comparison to core-shell microspheres, thereby facilitating a more seamless injection procedure and mitigating the risk of catheter occlusion throughout the operation.

### Evaluation of *in vitro* occlusion performance of prepared microspheres

To accurately replicate the embolization effect of three microspheres with different crosslink structures *in vivo*, we designed a three-dimensional vascular model made of stainless steel and polymethyl methacrylate (PMMA) with variable inner diameters (from 2.0 to 0.5 mm, shown in Supplementary Figure S3) and a constant flow rate (35 cm/s) to simulate a standardized physiological hemodynamic environment. This model incorporated the distribution of blood vessels observed in HCC patients while considering factors such as vascular tortuosity, as shown in Figure 3C. Two milliliters of stained microspheres mixed with the contrast medium were slowly injected into the vascular model. Normal saline was injected into the vascular model at a consistent rate to simulate the effect of blood on the embolic microspheres.

There are three typical embolism scenarios as displayed (Figure 3D). In condition I, the microspheres are more concentrated and relatively denser in the proximal vessel. This is often observed with softer embolic particles, as they are easily compressed and deformed, causing them to clump together in the proximal vessel. It gets to be challenging for the embolic agent to reach the distal vessel, leading to poor effectiveness of embolization at the distal end. As the saline-simulated rinsing persisted, the embolic microspheres are more compressed and move toward the distal artery. This corresponds to a clinical embolic phenomenon, accompanied by partial recanalization of the proximal vessel. In condition II, the embolic particles exhibit early intermittent embolization, with inadequate occlusion of certain areas of the artery. It frequently occurs when stiff embolic particles are unevenly distributed due to their limited elasticity. Through saline washing out, the microspheres in the proximal vessel progressively move toward the distal end, and the voids are gradually eliminated. Ultimately, recanalization in the proximal end occurs as well. In condition III, the embolic microspheres are uniformly and systematically positioned within the vessel immediately after the embolism procedure. The microspheres exhibit negligible migration and compression during flushing.

In our *in vitro* experiments, no noticeable alteration was detected in the alignment of the microspheres before and after the simulated blood test, which demonstrated a superior embolic effect in clinical practice. Homo microspheres had a lower crosslink degree, resulting in a comparatively soft texture, close to condition I. Core-shell microspheres had higher stiffness due to the high crosslink degree of their outer layer, trending to appear in condition II. In contrast, GCL microspheres showed exceptional elasticity, which was advantageous for condition III. The designed simulated artery model was capable of efficiently assessing the microsphere alignment in the vessel, providing valuable assistance for investigating the occlusion performance of synthetic microspheres. GCL microspheres exhibited the best occlusion performance in the test among the three microspheres (Figure 3E), which aligned with the outcomes of the previous mechanical properties analysis.

### Assessment of embolization efficacy of microspheres in a porcine kidney model

**Figure 4:**
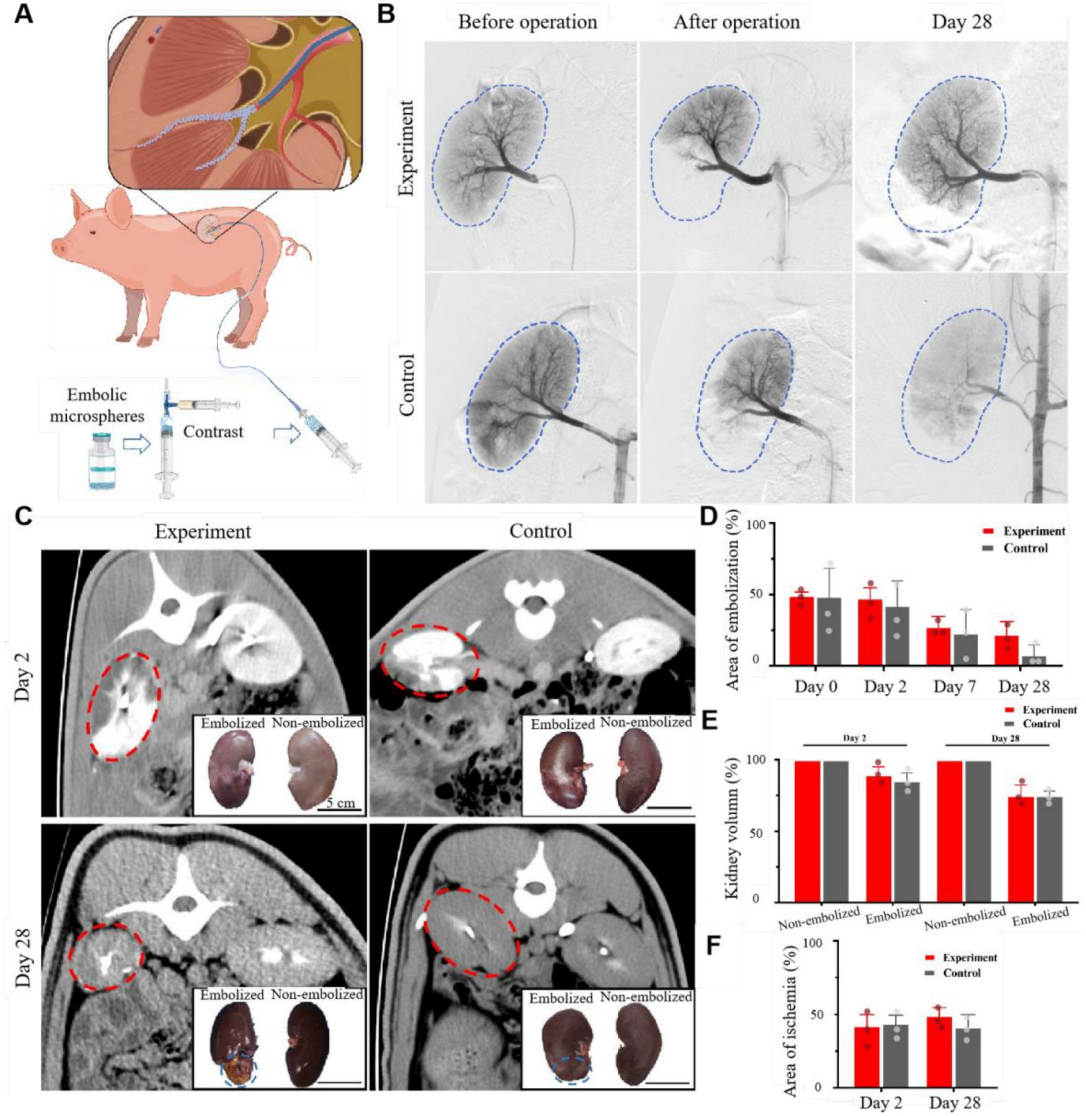
Evaluation of embolization efficiency in a porcine kidney model. (**A**) Schematic diagram of the animal experiment. (**B**) DSA images of the kidneys corresponding to pre-TACE and different post-TACE time points. Experiment group—GCL microspheres, control group—Embosphere® microspheres. The areas within the blue dashed line indicate the intact kidney region. (**C**) Cross-sectional plain CT images at different follow-up times and kidney photographs (scale bar 5 cm). Areas within red and blue dashed lines indicate intact and necrotic kidney regions, respectively. (**D**) Statistics on the embolized area at different follow-up times. (**E**) Statistics on the volume of kidney corresponding to 2 and 28 days post-TACE. (**F**) Statistics on ischemic area of the kidney on Day 2 and Day 28 post-TACE. There was no significant difference in the data of **D**, **E** and **F** between the two groups (*n *= 3) for most of the time points, indicating a comparable embolic effect.

To carry out a comprehensive assessment of the safety and effectiveness of the prepared microspheres, we conducted an *in vivo* embolism evaluation using the Bama pig kidney model (Figure 4A), which is the most commonly used model to evaluate the embolic effect of embolic agents [10]. The prepared GCL microsphere was utilized as the experimental group, while a commercially available embolic PVA microsphere named Embosphere with a basical homostructure was applied as the control group. During the embolization procedure, the microspheres in the experimental group exhibited better suspension properties and were able to flow through the microcatheter without any difficulty. The smoothness of the experimental group was not significantly different from that of the control group. All pigs in the minimally invasive embolization trials underwent the embolization without exhibiting any symptoms of suffering. The embolic microspheres were easily injected into every animal, and no clotting or catheter blockage was noticed. There were no complications observed during the surgery and follow-ups. All animals successfully survived until their predetermined time of sacrifice and maintained their ideal condition for the entirety of their follow-up period.

We carried out DSA of the two groups at distinct follow-up intervals. Both groups exhibited similar efficacies (Figure 4B). Prior to TACE, the shape and structure of the kidney, including the main artery and its branches, were clearly visible, exhibiting a naturally smooth appearance and uniform staining of the tissue. In certain regions at the lower pole of the right kidney post-embolization, renal parenchyma exhibited either no staining or minimal staining, and some distal vessels were not visible. Follow-up angiography indicated that a limited number of symptoms of proximal vessel recanalization were observed on Day 2 and Day 7 postsurgery in both groups. There was no angiographic evidence of nontarget embolization. Additionally, recanalization signs were observed in some distal vessels on Day 28 after surgery, and this phenomenon became increasingly evident as time progressed. The area of the renal embolic region in the DSA images revealed that the recanalization fraction in the control group was slightly higher than the experimental group or the embolization fraction in the experimental group was slightly higher than the control group. Hence, the embolism in the experimental group persisted for a longer duration.

We also performed computed tomography (CT) on Day 2 and Day 28 postoperation (Figure 4C). Both experiment and control groups exhibited irregular, low-density areas in the renal cortex suspected to be infarcted after TACE. Meanwhile, the kidneys were adherent to the perirenal tissues, and the boundaries between them was indistinct. The volume of kidneys was smaller than the contralateral kidneys after a longer follow-up period. The morphology of the embolized kidneys remained irregular, but there was significant improvement in the adhesion of the kidneys to the perirenal tissues. This aligns with the findings of the gross anatomy of the kidney.

The surface of the lower pole of the right kidney in the experimental group and control group showed primarily ischemic changes (e.g. necrosis, congestion, peritoneal exudation) at the 2-day follow-up, mottled with dark red on the surface and scattered hemorrhagic spots, and in some of the animals, there were obvious areas of bruising in the region of the embolism area, with a yellowish coloration visible on the surface, and some of the peritoneal adherence of the kidneys was severe. By the 28-day follow-up, the lower pole kidneys primarily exhibited atrophy, hardening with an uneven surface, nodular firmness upon palpation, and basically no adhesion to the surrounding tissues. The statistical analysis of kidney volumes (Figure 4D–F) based on the CT images and gross anatomy images revealed no significant disparity between the experimental group and control group in terms of the ischemia areas of the kidney at either the 2-day follow-up or 28-day follow-up time point, indicating comparable effectiveness.

### Histological evaluation

**Figure 5:**
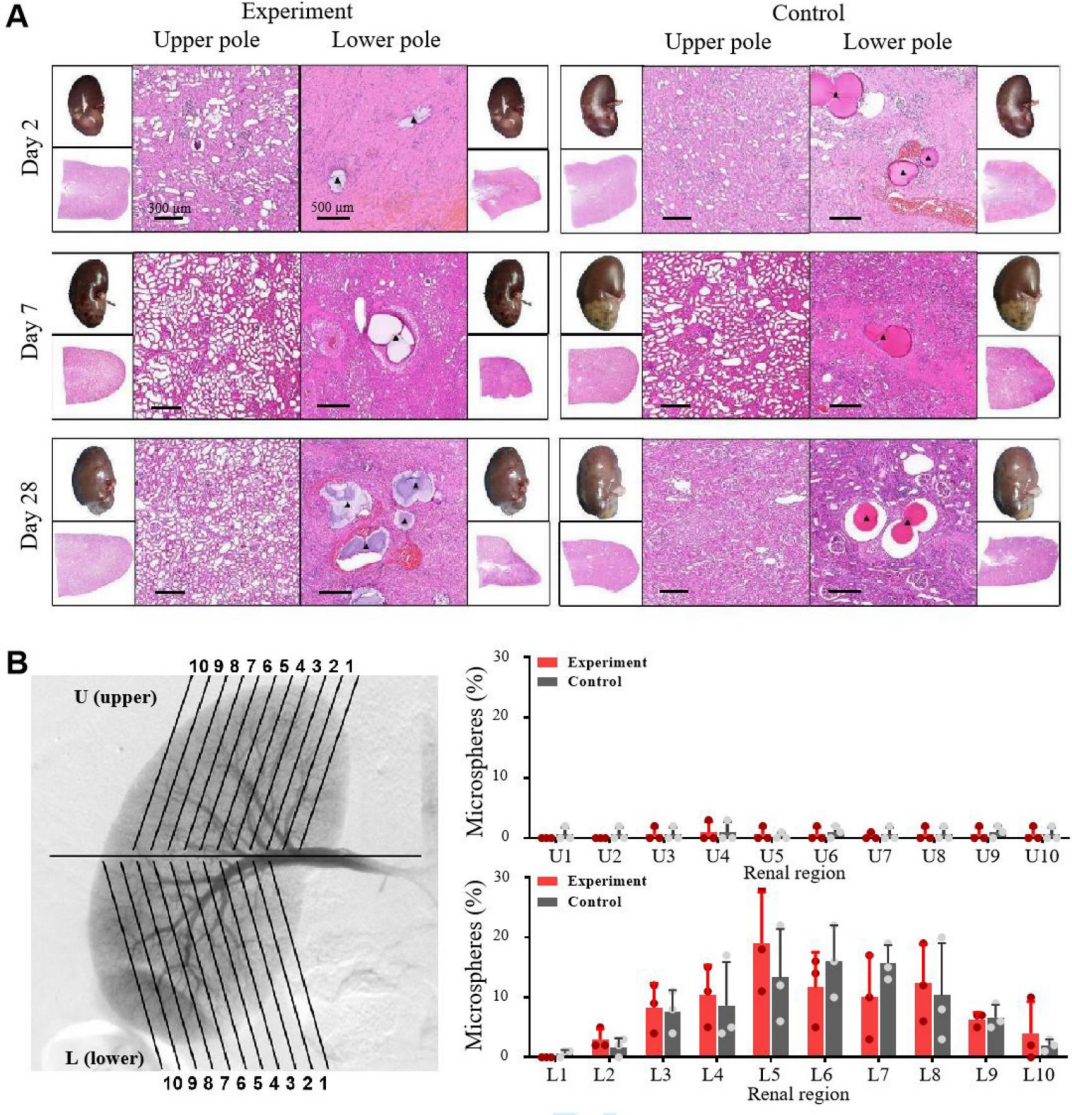
Histological evaluation of microspheres in porcine kidney. (**A**) Optical micrographs of slices of porcine kidney stained with HE. The upper pole (nontargeted embolization area) is shown to enable visualization of inflammatory cell infiltration or off-target microsphere distribution; the lower pole (target embolizationarea) is shown to facilitate the morphological details of residual microspheres, where ▲marks the local regions with microspheres. (**B**) Pathologic sections of DSA images and distribution of microspheres in porcine kidney post-TACE.

Animals were histologically analyzed after sacrificed. Two days postoperatively, both the experiment and control groups exhibited significant necrosis in the renal cortex, partial necrosis in the medulla, and extensive hemorrhage and congestion (Figure 5A). Moreover, damage to arteries at the embolization site was observed, with necrotic cells and a small quantity of inflammatory cells seeping from the perivascular region. On Day 7 postoperatively, the observations indicated the absence of glomerular and tubular structures, the presence of inflammatory cells infiltrating the kidneys, and the calcification of necrotic areas. By Day 28, both groups exhibited tissue shrinkage at the lower pole of the kidney where the embolism occurred. There was the development of granulation tissue around the embolism site, infiltration of inflammatory cells, an increase in collagen fibers in the fibrotic tissue, and the formation of numerous small abnormal blood vessels. Furthermore, calcification with the deposition of lipofuscin was observed in the outer layer of the kidney. Arterial embolization caused tissue damage, which was evidenced by tissue ischemia, vascular injury, necrosis and inflammatory responses. While both the experiment and control groups exhibited similar pathology outcomes, the experimental group suggested a relatively greater extent of necrosis at the embolic location, indicating a better embolic outcome.

Pathological analysis was performed according to the location of Figure 5B for zonal sampling, and the number of embolic microspheres in each zone was counted. Both groups showed roughly normal distribution in the region of L1–L10. The distribution of the experimental group in the proximal region was greater than that of the control group, aligning with the embolic region on the DSA images. This indicated that the experimental group was able to maintain a more effective embolization, with a low probability of recanalization or a slow rate of recanalization. The statistical analysis of the upper pole of the right kidney revealed a minimal presence of microspheres in this area. This can be attributed to the presence of shared vascular branches between the upper and lower poles, making it challenging to avoid during the surgical procedure. The majority of the microspheres remained primarily concentrated at the intended target site.

The safety of embolic microspheres is crucial as a medical device. Gross anatomical and pathological results showed that, except for the kidneys, the major organs, such as the heart, liver, spleen, lungs, gallbladder and brain, showed no obvious change and no significant abnormality during the 28-day follow-ups (data not shown). In addition, the animals were monitored for blood counts, coagulation function indexes and liver and kidney function indexes during the follow-up. The blood indices of experimental microspheres and control microspheres were similar at each follow-up point after the procedure, and no significant difference was seen in Supplementary Table S2, illustrating that the embolization basically had no adverse reaction to the blood indices of the animals. In each group of animals, there was no significant difference in most of the indicators when compared with the preoperative indicators; while there were significant differences in urea and glutamine transaminase in some groups, they were basically within the normal range. Hence, the experimental microspheres and control microspheres had equivalent safety.

### Clinical case of GCL microspheres

**Figure 6:**
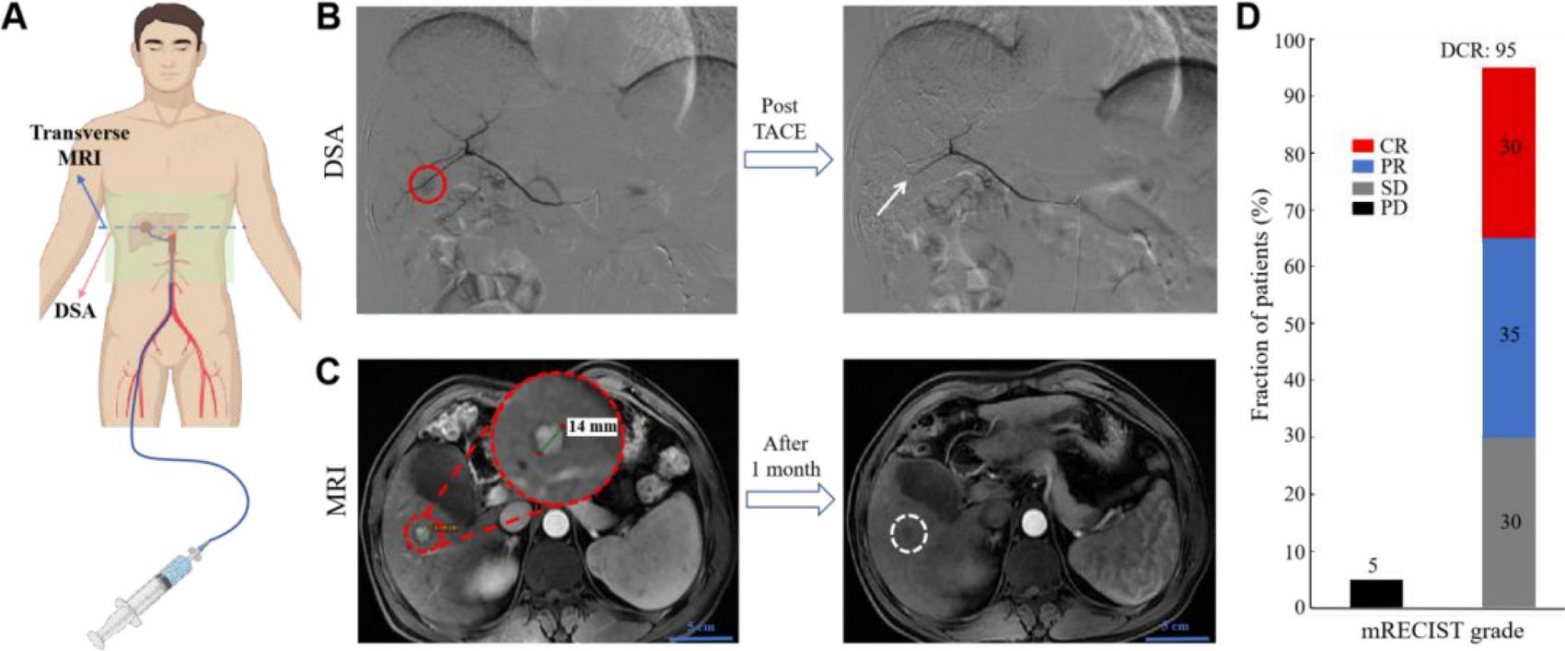
Clinical trial results. (**A**) Scheme of clinical case utilizing WizSphere® for TACE treatment of HCC. (**B**) a representative case of 51-year-old male: DSA image before and post-embolism. The red cycles show the lesion location. (**C**) Transverse MRI of the lesion before TACE treatment and 1-month follow-up MRI (baseline tumor diameter 14 mm, scale bar 5 cm). (**D**) Clinical cases statistical data at 3-month follow-up (20 cases). The *X*-axis indicates the tumor category after TACE treatment by mRECIST standard (Supplementary Table S5), and the *Y*-axis represents the fraction of each category relative to the total number of operation patients. CR, complete response; PR, partial response; SD, stable disease; PD, progressive disease; DCR, disease control rate (CR + PR + SD).

As permitted, the novel GCL microsphere (WizSphere®) was used to treat HCC in a 51-year-old male patient whose Child-Pugh classification (Supplementary Table S3) was A and ECOG (Supplementary Table S4) performance status was 0. The corresponding TACE procedure is shown in Figure 6A. The catheter was inserted through the right femoral artery and ultimately selected to the site of a liver tumor. Subsequently, chemotherapeutic agents and the GCL microspheres were injected gradually into the lesion to achieve therapeutic effects.

According to the DSA images, the blood supply to the stained tumor was interrupted, for no signal was detected 5 min after the embolism, suggesting the good outcome of instantaneous embolization (Figure 6B). After review at 1-month post-TACE, no secondary embolization was performed for this patient. Prior to TACE, the largest lesion-located segment 5 with 13.9 mm diameter, was presented in MRI (Figure 6C).

Furthermore, the statistical outcomes of TACE treatments using GCL microspheres in a cohort of 20 patients are shown in Figure 6D. The proportion of patients achieving complete response (CR) or partial response (PR) reached 65% according to the mRECIST (Supplementary Table S5), which is defined as the absence of any intra-tumoral arterial enhancement in all target lesions at 1 month [11]. The DCR of this cohort is 95%, much better than the average embolization [12]. No major adverse events, such as hepatic insufficiency, severe ectopic embolism, severe bone marrow suppression, occurred during or after implantation. Patients have not experienced any serious adverse events and recovered uneventfully so far. The assessment of lesion size variation was conducted by follow-up MRI, including the chest, abdomen and pelvis. The lesion was identified as considerably shrunk. This proved the successful embolization of the tumor blood supply, which resulted in necrosis of the tumor. For the six CR patients, their liver function, blood clotting and serum alpha fetoprotein (AFP) were basically normalized (Supplementary Table S6), indicating that the operation was successful. Hence, the GCL microspheres exhibit positive therapeutic effectiveness in TACE therapy.

## Discussion

With the development of human society, implantable medical devices are expected to have higher performance and better safety [13–15]. Many high-performance materials have been reported and partially applied in medical devices [16–26]. Owing to their compatibility with human tissues, etc., hydrogels have recently been paid much attention as medical materials [27–39], and the embolization particles for TACE belong to a microhydrogel. TACE is the preferred therapeutic approach for patients with intermediate-stage HCC, namely, Barcelona Clinic Liver Cancer (BCLC) stage B [40, 41]. It is of great significance to develop the synthesis method of high-performance embolized microspheres and carry out mechanism research [42–50].

Hydrogel microspheres with well-defined structures and precise particle sizes can be produced using suspension polymerization. With the usage of different materials, the resulting microspheres exhibit different physical properties, including hydrophilicity, surface tension, ionic charge and roughness [50]. This leads to variation in the compressibility and elasticity of microspheres, subsequently affecting the deliverability of the prepared embolic microspheres and their distribution within specific vascular networks [51]. Embolic microspheres generally require high compressibility to enhance their deliverability. Previous literature has indicated that particles with higher compressibility exhibit greater deformability, leading to an increased probability of distal embolism [41]. Nevertheless, sufficient elasticity is also essential, enabling the microspheres to rapidly recover their original size upon exiting the microcatheter. Additionally, the diameter of the microspheres impacts their intravascular distribution, thereby affecting the embolic performance and embolic predictability. Optimal embolic microspheres need a balance between compressibility and elasticity. Unfortunately, high compressibility and high elasticity seem to be impossible to achieve simultaneously in a homogeneous gel consisting of a single substance. The introduction of composite-structured microspheres was proposed to resolve this dilemma. Microspheres with a core-shell structure typically consist of a shell part and a core part made from materials with distinct compositions or structures. An increase in the crosslink density of the gel results in a more compact three-dimensional network structure and reduced room for water molecules, leading to diminished dissolving rates and decreased flexibility [37]. Furthermore, an increase in the crosslink density of the hydrogel constrains the mobility of the macromolecular chains inside the matrix, enhancing the mechanical strength. Therefore, integrating a core characterized by a reduced crosslink degree and enhanced hydrophilicity with a strongly crosslinked shell is a feasible option for achieving a balance between compressibility and elasticity. Nevertheless, combining two layers with different mechanical strengths is subject to stress concentration. Herein, we present a one-step gradient crosslink synthesis strategy that successfully generates the microsphere with a gradient core-shell microstructure. The core of the manufactured microsphere exhibits low crosslink density and a larger proportion of hydrophilic AMPS, resulting in a loose internal structure with high water content that is beneficial for deformation. The increased crosslink density and raised proportion of hydrophilic NAAADA in the shell resulted in a denser three-dimensional network nearer to the outer shell, and such a gradient favors the strength of the whole microsphere. The shell of the prepared microsphere has higher crosslink density and a larger proportion of hydrophilic NAAADA, offering the ability to maintain the integral shape of the microsphere morphology. Hence, the gradient microspheres might exhibit an excellent elastic fraction in principle.

We assessed the effect of the microsphere structure on the mechanical property by comparing the mechanical characteristics of microspheres with three distinct microstructures prepared with different synthesis steps. The unique geometric configuration of embolic microspheres demanded the use of compression testing to evaluate their mechanical characteristics. The homogeneous microspheres had the least rigidity and relaxation force. The uniform distribution of the homogeneous crosslink agent resulted in a low average crosslink density under a given quantity of the agent. The free movement between molecular chains was relatively greater, and the interaction force between molecular chains was smaller. Relative displacement was easy to occur under the action of external forces, then its compression strength was low. The traditional core-shell microspheres produced with the two-step process exhibited high rigidity but low relaxation force. Their highly crosslinked shells offer robust support, enhancing compressive strength. However, the weak interface between the core and shell caused uneven external force transfer, resulting in local stress concentration and structural damage. Moreover, the ineffective transfer of internal force during recovery led to reduced relaxation force. The rigidity of GCL microspheres was between those of homogeneous microspheres and core-shell microspheres, and the relaxation force of GCL microspheres was significantly higher than that of core-shell microsphere. Shell with a high crosslink density offered adequate support under compression, and the gradient core-shell structure reduced stress concentration, thereby improving elasticity.

As the mechanism is concerned, the differences among homogeneous, core-shell and gradient microspheres may arise from their network architectures and stress transfer pathways. Homogeneous microspheres exhibit uniform crosslinking density, leading to isotropic but brittle failure without crack arresting. Core-shell microspheres suffer from sharp mechanical mismatch at the discrete core-shell interface [52], causing severe stress concentration, interfacial delamination and premature failure under load [30]. In contrast, gradient microspheres feature a continuous variation of crosslink densities from the soft core to the stiff shell. A gradient architecture may eliminate the abrupt boundary, enabling smooth stress transfer through an intermediate transition zone [53]. The spatially varying network introduces multiple energy-dissipation mechanisms, including sequential polymer chain rearrangement, non-affine deformation of the loose core and progressive resistance to crack propagation, yielding synergistic enhancement of compressive strength, recoverability and fatigue resistance. The superiority of the gradient design stems from tightly integrated interfacial bonding formed by shell-forming precursors penetrating and polymerizing within the superficial core layers, creating an interpenetrating network with molecular-level interlocking. This seamless transition distributes mechanical stress uniformly, prevents microcrack initiation and ‘stitches’ the core and shell together with adhesion far exceeding simple surface attachment. Consequently, the absence of a discrete weak interface allows the microsphere to endure repeated compressive cycles with minimal internal energy dissipation, maintaining structural integrity throughout procedural manipulation and the post-embolization hemodynamic environment.

## Conclusion

A type of embolic microspheres with the GCL core-shell structure were synthesized on an industrial scale. Owing to their distinctive structure, the GCL microspheres achieved an optimal equilibrium of strength and resilience. *In vitro* study demonstrated that these microspheres possess enhanced penetration into distal blood vessels, more uniform distribution, reduced risk of migration and minimal proximal recanalization over time, resulting in optimal embolic efficacy. The *in vivo* study was conducted in a porcine kidney model. The GCL microspheres exhibited good efficacy in terms of the area of embolic necrosis, the area of microsphere distribution and the degree of necrosis of the pathological tissues. The clinical trials conducted further validated the safety and embolic effect of the produced microspheres in humans. This provides more therapeutic options with clinicians and is expected to have a broad application prospect in the future in diverse clinical settings, including the treatment of hypervascular tumors like liver cancer, as well as other conditions such as uterine fibroids and prostate embolization. This article reports a method for producing gradient-crosslinked embolic microspheres and reveals the relationship between mechanical properties and embolic efficiency, which is stimulating for the development of embolic agents.

## Supplementary Material

rbag149_Supplementary_Data
