# Supplementary material for: A gradient core-shell microsphere to enable effective embolization of liver cancer via interventional therapy
Source: Regen Biomater. 2026 Jul 22;13:rbag149. doi: 10.1093/rb/rbag149 (PMC13466941; doi:10.1093/rb/rbag149)
Supplement: Supplementary file 1 [file rbag149_supplementary_data.zip › Gradient core-shell microspheres SI-20260703.docx]

Supporting Information

**A gradient core-shell microsphere to enable effective embolization of liver cancer via interventional therapy**

Hongtao Sun^1, 2^, Lijuan Yao^2^, Xin Wang^1^, Hao Xu^3^, Chaozhou Li^2^, Xin Li^2^, Lin Yu^1^, Haibo Che^2^, Peng Sun^2^*, Yinghua Zou^4^*, Jiandong Ding^1^*

^1^State Key Laboratory of Molecular Engineering of Polymers, Department of Macromolecular Science, Fudan University, Shanghai 200438, China

^2^China Cardiolink Science (Shenzhen) Medical Technology Development Co., Ltd, Shenzhen 518122, China

^3^China Interventional Radiology Department, The Affiliated Hospital of Xuzhou Medical University, Xuzhou 221004, China

^4^Department of Interventional Radiology and Vascular Surgery, Peking University First Hospital, Beijing 100034, China

*Correspondence authors.

**Jiandong Ding**, Email: [jdding1@fudan.edu.cn](mailto:jdding1@fudan.edu.cn)

**Yinghua Zou**, Email: [13801105222@139.com](mailto:13801105222@139.com)

**Peng Sun**, Email: amanda.sun@cardiolink.com.cn

**Figure S1.** Histogram of the diameter distributions for the five available size ranges for gradient crosslinking (GCL) microspheres. Microspheres were sized from microscope images.

**Figure S2.** Water contents of three types of microspheres. There was no significant difference between any two of the three groups (*n* = 3 per group).**.**

**Figure S3.** Scheme of embolism assessment model. R2.0, 1.5, 0.8, 0.5 denote the radii of the marked catheters with 2.0, 1.5, 0.8 and 0.5 mm, respectively.

**Table S1.** Minimum passable microcatheters corresponding to different microspheres (300-500 μm).

|  | Homo microsphere | Core-shell microsphere | GCL Microsphere |
| --- | --- | --- | --- |
| Minimum size of microcatheter that allows the microsphere to pass through | 1.8F | 2.2F | 1.8F |
| Inner diameter and length of the microcatheter | 0.43 mm  *150 cm | 0.53 mm  *150 cm | 0.43 mm  *150 cm |

**Table S2.** Blood routine, coagulation function indexes and liver function indexes in experiment group and control group.

| Criteria | Experiment  (2 D) | | Control  (2 D) | | Experiment  (7 D) | | Control  (7 D) | | Experiment  (28 D) | | Control  (28 D) | |
| --- | --- | --- | --- | --- | --- | --- | --- | --- | --- | --- | --- | --- |
|  | [initial](https://www.youdao.com/w/initial/#keyfrom=E2Ctranslation) | 2 D | [initial](https://www.youdao.com/w/initial/#keyfrom=E2Ctranslation) | 2 D | [initial](https://www.youdao.com/w/initial/#keyfrom=E2Ctranslation) | 7 D | [initial](https://www.youdao.com/w/initial/#keyfrom=E2Ctranslation) | 7 D | [initial](https://www.youdao.com/w/initial/#keyfrom=E2Ctranslation) | 28 D | [initial](https://www.youdao.com/w/initial/#keyfrom=E2Ctranslation) | 28 D |
| **WBC**（x10^9^/L） | 13.4  ±1.4 | 11.9  ±0.2 | 12.5  ±1.6 | 13.3  ±4.0 | 7.9  ±2.7 | 6.1  ±0.2 | 8.8  ±3.9 | 8.2  ±3.1 | 24.2  ±2.9 | 21.4  ±2.3 | 23.2  ±4.7 | 18.6  ±9.3 |
| **RBC**（x10^12^/L） | 7.3  ±0.3 | 7.1  ±0.7 | 7.4  ±1.1 | 6.3  ±1.3 | 5.4  ±0.5 | 5.8  ±0.9 | 5.6  ±0.7 | 5.1  ±0.1 | 7.5  ±0.6 | 7.7  ±0.2 | 6.5  ±0.9 | 8.5  ±0.8 |
| **HGB**  （g/L） | 136.3  ±6.3 | 132.3  ±9.3 | 133.0  ±9.2 | 114.0  ±14.2 | 105.3  ±16.7 | 111.0  ±23.3 | 112.7±26.4 | 103.7±12.3 | 143.0±14.8 | 147.0  ±9.4 | 121.3  ±5.7 | 160.0  ±19.1 |
| **PLT**（x10^9^/L） | 416.7  ±32.6 | 335.7  ±75.6 | 375.0  ±114.4 | 291.3  ±66.2 | 352.0  ±27.6 | 391.3  ±16.4 | 401.3  ±49.1 | 431.7  ±93.6 | 298.7  ±81.1 | 356.0±153.2 | 357.7  ±85.2 | 302.0  ±74.5 |
| **PT**(sec) | 9.4  ±0.4 | 9.1  ±0.8 | 8.3  ±0.3 | 9.5  ±0.9 | 9.8  ±0.6 | 9.0  ±0.2 | 9.7  ±0.1 | 8.2  ±5.8 | 9.2  ±1.3 | 8.5  ±0.4 | 8.9  ±1.3 | 8.9  ±0.5 |
| **APTT**(sec) | 10.6  ±3.9 | 11.5  ±5.0 | 8.8  ±3.3 | 9.3  ±3.0 | 7.6  ±1.8 | 5.3  ±1.5 | 7.7  ±1.0 | 9.9  ±10.5 | 8.4  ±3.1 | 6.4  ±2.5 | 7.4  ±1.3 | 6.3  ±3.2 |
| **FIB** (g/l) | 1.0  ±0.2 | 1.5  ±0.8 | 2.0  ±0.6 | 1.4  ±1.2 | 1.0  ±0.4 | 2.4  ±1.4 | 1.2  ±0.4 | 2.1  ±0.5 | 2.1  ±0.8 | 1.5  ±0.3 | 2.0  ±0.4 | 1.1  ±0.5 |
| **TT** (sec) | 19.1  ±1.9 | 19.0  ±1.5 | 18.9  ±3.1 | 17.8  ±2.2 | 34.9  ±4.2 | 29.3  ±4.9 | 41.0  ±1.0 | 62.3  ±34.5 | 28.2  ±11.4 | 19.7  ±1.5 | 26.4  ±8.7 | 20.8  ±3.0 |
| **ALT**/**AST** | 1.1  ±0.1 | 0.67  ±0.12 | 1.1  ±0.5 | 0.90  ±0.65 | 1.2  ±1.1 | 0.77  ±0.73 | 0.97  ±0.56 | 0.53  ±0.48 | 2.3  ±1.1 | 1.9  ±1.0 | 2.4  ±0.7 | 1.2  ±0.6 |
| **ALP**（U/L） | 105.0±  26.2 | 89.3  ±24.5 | 70.7  ±24.7 | 215.0  ±164.1 | 33.3  ±13.3 | 68.3  ±9.6 | 52.7  ±25.6 | 62.7  ±28.0 | 94.3  ±33.8 | 75.0  ±31.8 | 97.0  ±41.2 | 51.3  ±30.1 |
| **TBIL**（umol/L） | 0.73  ±0.19 | 0.67  ±0.12 | 1.1  ±0.4 | 1.1  ±0.5 | 0.67  ±0.66 | 0.80  ±0.73 | 0.80  ±0.85 | 0.13  ±0.09 | 0.47  ±0.31 | 0.43  ±0.19 | 0.43  ±0.09 | 0.73  ±0.34 |
| **GGT**（U/L） | 42.0  ±2.5 | 60.0  ±19.0 | 48.3  ±35.3 | 59.0  ±19.3 | 56.7  ±4.8 | 29.3  ±24.8 | 43.7  ±9.9 | 27.3  ±17.3 | 26.0  ±15.0 | 25.3  ±15.2 | 35.3  ±21.9 | 33.3  ±22.5 |
| **UREA**（mmol/L） | 2.2  ±0.7 | 3.9  ±0.4 | 5.1  ±2.6 | 5.9  ±2.6 | 4.2  ±0.9 | 6.5  ±3.3 | 3.1  ±1.3 | 5.1  ±3.8 | 4.7  ±2.2 | 6.2  ±5.0 | 4.7  ±3.0 | 6.1  ±4.7 |
| **CRE**（umol/L） | 79.7  ±7.6 | 91.3  ±6.8 | 83.7  ±13.5 | 109.7  ±39.1 | 80.0  ±20.6 | 75.7  ±25.7 | 71.7  ±24.1 | 56.7  ±35.1 | 80.7  ±20.4 | 82.0  ±25.7 | 73.7  ±23.2 | 77.0  ±26.0 |

**Note: WBC－white blood cell, RBC－red blood cell, HGB－hemoglobin, PLT－platelet, PT－prothrombin time, APTT－activated partial thromboplastin time, FIB－fibrinogen, TT－thrombin time, ALT－alanine aminotransferase, AST－aspartate aminotransferase, ALP－alkaline phosphatase, TBIL－total bilirubin, GGT－γ-glutamyl transferase, UREA－urea, CRE－creatinine.**

**Table S3.** Child-Pugh classification.

| Criteria | 1 point | 2 points | 3 points |
| --- | --- | --- | --- |
| Encephalopathy | None | Grades 1 and 2 | Grades 3 and 4 |
| Ascites | None | Slight | Moderate |
| Serum bilirubin (mg/dL) | <2 | 2-3 | >3 |
| Serum albumin (g/dL) | >3.5 | 2.8-3.5 | <2.8 |
| Prothrombin time (sec.) | <4 | 4-6 | >6 |

Note: Child-Pugh A **－** 5-6 points, Child-Pugh B **－** 7-9 points, Child-Pugh C **－** 10-15 points.

**Table S4.** Eastern Cooperative Oncology Group (ECOG) classification.

| Grade | ECOG performance status |
| --- | --- |
| 0 | Fully active, able to carry on all pre-disease performance without restriction |
| 1 | Restricted in physically strenuous activity but ambulatory and able to carry out work of a light or sedentary nature |
| 2 | Ambulatory and capable of all selfcare but unable to carry out any work activities; up and about more than 50% of waking hours |
| 3 | Capable of only limited selfcare; confined to bed or chair more than 50% of waking hours |
| 4 | Completely disabled; cannot carry on any selfcare; totally confined to bed or chair |
| 5 | Dead |

**Table S5.** Modified response evaluation criteria in solid tumors (mRECIST) for hepatocellular carcinoma (HCC)

| Grade | Description |
| --- | --- |
| CR | Disappearance of any intratumoral arterial enhancement in all target lesions |
| PR | At least a 30% decrease in the sum of diameters of viable (enhancement in the arterial phase) target lesions, taking as reference the baseline sum of the diameters of target lesions |
| SD | Any cases that do not qualify for either partial response or progressive disease |
| PD | An increase of at least 20% in the sum of the diameters of viable (enhancing) target lesions, taking as reference the smallest sum of the diameter of viable (enhancing) target lesions recorded since treatment started |

Note: CR **－**complete response, PR **－**partial response, SD **－** stable disease, and PD **－** p**rogressive disease of** **mRECIST set by American Association for the Study of Liver Diseases (AASLD).**

**Table S6.** Liver function, blood clotting and serum alpha fetoprotein (AFP) data of CR patients (*n* = 6).

|  | Measured Mean Value | Reference Value |
| --- | --- | --- |
| AFP (ng/ml) | 0.05 | <10 |
| GPT (IU/L) | 41.4 | ≦40 |
| GOT (IU/L) | 40.2 | ≦40 |
| TBIL | 18.3 | 3.4-17.1 |
| ALP (IU/L) | 89.5 | 40-129 |
| PT (s) | 13.8 | 11-14 |

**Note: AFP－alpha fetoprotein, GPT－glutamate pyruvate transaminase, GOT－glutamate oxaloacetate transaminase, TBIL－total bilirubin, ALP－alkaline phosphatase, PT－prothrombin time.**
